# IDcare – a longitudinal register study of pre-pandemic and pandemic health care utilization and diagnostic profiles among people with intellectual disabilities in southern Sweden

**DOI:** 10.1007/s10654-024-01151-3

**Published:** 2024-09-23

**Authors:** Magnus Sandberg, Jimmie Kristensson, Anna Axmon

**Affiliations:** 1 Department of Health Sciences, Lund University, Lund, Sweden; 2 Institute for Palliative Care, Lund University and Region Skåne, Lund, Sweden; 3 Epidemiology, Population Research, and Infrastructures at Lund University, Lund University, Lund, Sweden

**Keywords:** Intellectual disabilities, Healthcare utilisation, Diagnosis, Registers

## Abstract

The aim of the creation of this cohort was to investigate patterns of health and health care utilisation before and during the COVID-19 pandemic, overall and in relation to specific diagnoses, among people with intellectual disabilities (ID) compared to the general population. People living in Skåne, the southernmost region of Sweden, on 1st of January 2014 with at least one diagnosis of ID (ICD-10 codes F70-F79) or Down syndrome (DS; Q90), or support and/or services according to the LSS act comprised the ID cohort (*n* = 14 716). People living in the same family and/or household as a person in the ID cohort constituted the ID family cohort (*n* = 31 688), and those remaining comprised the general population cohort (gPop; *n* = 1 226 955). Data has been collected for all three cohorts from several national and regional registers. These include registers for health care (2014–2021), deaths (2014–2021), COVID-19-related health care (vaccinations, intensive care, palliative care, 2020–2021). The prevalence of ID was 1.2%. In the ID cohort, 77.9% had at least one measure of support, 5.8% at least one Q90-diagnosis and 63.8% had at least one F7-diagnosis (26.9% mild (F70), 7.4% moderate (F71), 2.8% severe (F72), 1.4% profound (F73), and 25.4% other/unknown (F78/F79)). Compared to the gPop there were more people in the younger age groups in the ID cohort. At this point, no additional collection of data will be carried out. However, there is a possibility to add data from the registers to include years after 2021 or from additional registers. Future publications will explore relevant research questions and report key findings in relation to health among people with ID. Future results will be used to inform policy and practice on people with ID.

## Introduction

The definition of intellectual disabilities (ID), in some countries referred to as intellectual and developmental disabilities (IDD), mental retardation or learning disabilities, may vary slightly. According to the World Health Organization (WHO), ID is “a condition of arrested or incomplete development of the mind, which is especially characterized by *impairment of skills manifested during the developmental period*,* skills which contribute to the overall level of intelligence*,* i.e. cognitive*,* language*,* motor*,* and social abilities*” [1]. Similar definitions are provided by WHO: s International Classification of Functioning, Disability and Health (ICF) [2] and the American Association on Intellectual and Developmental Disabilities (AAIDD, former American Association on Mental Retardation, AAMR) [3]. However, these also include concurrent deficits in adaptive behaviour functioning. Commonly, ID is considered to have onset before the age of 18 years of age. However, the AAIDD definition allows for ID to originate until the age of 22 [3]. The severity of ID is usually considered at four levels, mild (IQ 50–69), moderate (IQ 35–49), severe (IQ 20–34), and profound (IQ < 20) [1]. Studies with samples based on total populations report a prevalence of challenging behaviour among people with ID of about 20% [4–6], and an association between severity of ID and challenging behaviour [4, 5]. Challenging behaviour may present itself in various ways, but one definition suggested by Einfeld and Emerson [7] as a “culturally abnormal behaviour(s) of such intensity, frequency or duration that the physical safety of the person or others is placed in serious jeopardy, or behaviour which is likely to seriously limit or deny access to the use of ordinary community facilities”.

Several studies have investigated the prevalence of ID. Among those that have used large samples based on national- or regional data, only a few have investigated the whole population of people with ID, and not only a certain age-group or a certain level of ID. This include studies from Taiwan [8], Ireland [9], Spain [10] Finland [11, 12], The Netherlands [13], Scotland [14] and Sweden [15], and report prevalences at 0.24–0.70%. A systematic review with a meta-analysis of 52 published papers estimated the global prevalence of ID to be 1.09%, with higher prevalence in males both in children/adolescent and adult populations [16]. A more recent review, however without meta-analysis, reported a prevalence of 0.05 to 1.55% [17]. Thus, the reported prevalence of ID seems to vary, probably because of different study designs, sampling procedures, settings, and operationalization of ID. As different methods for operationalization are likely to fail to identify certain groups of individuals with ID, it is likely beneficial to use several ways, to be able to get as comprehensive and representative sample as possible. Furthermore, it also makes it important to give a comprehensive description of cohorts used and to investigate the impact of the different sampling techniques.

People with ID demonstrate higher prevalence of a number of physical [18, 19] and psychiatric health conditions [20]. Given a more complex health, it may not be surprising that people with ID have been reported to have higher risks of unplanned somatic specialist in- and outpatient visits [21, 22] and visits to emergency departments [23–25]. Also for planned health care utilization there are differences between people with ID and the general population. Children, adolescents, and adult people with ID have been reported to have higher risks than the general population of for example primary care consultations [24] and visits to general practitioner [25, 26], consultations in general medicine [27], in- and outpatient specialist care [21, 22, 28] and psychiatric care [27, 29]. There are some exceptions, for example where older people with ID seem to use less planned care compared to the general population [21, 22, 28].

Medical and social advances, with a wide range of medical procedures and medications in combination with support and care for people with ID, that were not available fifty years ago are accessible today. As a result, remarkable improvements have been found during the last decades when it comes to, for example, average life expectancy [11, 30]. In Sweden, people with Down’s Syndrome (DS) had an annual increase in median age of death of 1.8 years between 1969 and 2003, with a median age of death of 60 years in 2003 [31]. Moreover, with the exception of people with DS or severe or multiple disabilities, people with ID now have a life expectancy similar to that of the general population [11, 30]. In contrast, Heslop, Blair [32] found that the median age at death among people with mild ID is still substantially lower than in the general population (68 vs. 78 years). Even so, the number of people with ID over the age of 60 is expected to increase from about 640 000 in 2000 to 1.2 million in 2030 [33]. Thus, today people with ID both survive previously fatal conditions, live with symptoms of diagnoses that are not treatable, and may develop other conditions due to normal aging. This has posed increased demand on health care and social services to meet the needs of this aging group of people with ID.

Also, when it comes to number of avoidable deaths were higher in people with ID compared to the general population [32, 34, 35]. Thus, despite these improvements in people with ID there are still inequities in life expectancies compared to the general population.

Although previous research on health care for people with ID have been published, many studies have limitations regarding for example small samples [36–38] and/or from single/smaller institutions [39–41]. This means that they are particularly sensitive to selection bias and with limited external validity as result. Furthermore, when looking at health care utilization in most cases, data from only one or few different health care providers, for example only primary care or ED visits, were used [42–44]. This may be a problem, as higher health care utilization at one provider could mean lower at another, or that a person without a registered diagnosis at one provider may have this registered at another. All in all, this means that to be able to draw conclusions about differences in health or health care needs among people with ID it is important to have larger samples from a certain area rather than a single care facility, and to collect data from a wider range of the health system.

Furthermore, most studies have rather small samples and are often lacking comparison group from the general population. Another issue is that many studies only include certain ages. As people with ID are a very heterogenous group, and with higher morbidity and mortality than the general population it is important to include people of all ages to be able to compare age groups throughout the life span. If not, there is a risk of selection bias in terms of healthy survivors. Investigating all ages longitudinally is probably more likely to facilitate a better understanding of health-related changes throughout the whole life span, as well as the ageing process in this group. Furthermore, using one single way of defining the sample could also be a risk of not including all people with ID in the sample.

To be able to address these shortcomings a new cohort, IDcare, was launched. After the initiation of this cohort COVID-19 pandemic struck, giving us the possibility to investigate the effects of the pandemic by expanding the study period. Both the original cohort and the expansion was funded by Forte, The Swedish Research Council for Health, Working Life and Welfare. This cohort will in various ways further the knowledge about health and health care utilization among people with ID compared to the general population before and during the COVID-19 pandemic.

The overall aims of IDcare are as follows:

1. To investigate patterns of health care utilization as a potential indicator of quality of care.
2. To assess the effects of the COVID-19 pandemic on access to health care among people with ID.

Both aims will be investigated comparing people with ID to the general population, in relation to severity of ID, as well as with respect to presence of behaviour impairment.

## Cohort description

### Study setting

IDcare was performed in Skåne (Scania), the southernmost of Sweden’s 21 counties. In 2022, the population of Skåne was approximately 1.4 million inhabitants, corresponding to 13% of the country’s total population [45]. Except for the Stockholm region, which includes the Swedish capital, Skåne was the most densely populated region in the country, with 120 inhabitants/km^2^ [45]. The region consists of both urban and rural areas and is dominated by agricultural land. There are 21 large localities with more than 10 000 inhabitants, but only two had more than 100 000 inhabitants, with Malmö as the biggest city with a population of about 300 000. About 24% of the inhabitants in 2022 were born outside Sweden, compared to 20% in the country as a whole [45]. The county council of Skåne is called Region Skåne.

Sweden has a welfare system and health care is mainly funded by taxes, with only small out-of-pocket costs. The system is decentralized where health care, treatment, rehabilitation, and specialized medical care in inpatient and outpatient settings or in primary care centres, as well as physician home care, are provided by 21 county councils and regulated by the Health Service Act [46]. This act is fundamental for equality reasons as it establishes the right to equal access to health care for all Swedish residents. Sweden has a long tradition in population-based registers with information on an individual level. These registers are compiled by government agencies or other organisations. Several cover the health care and services provided in Sweden. Those that are related to health care and services acts [46–48] are kept by The National Board of Health and Welfare and are used for analyses and development of health care and services [49]. Besides these, there is a range of Swedish National Quality Registers (NQR). These are usually initiated and maintained by professions in health care and cover a certain group of patients with a specific diagnosis or treated using a certain procedure, for example hip fractures or preventative care for elderly. These quality registers also contain data on an individual level and include patient problems, medical interventions, and outcomes after treatment within all health care production. However, whereas all health care is included in the registers at the National Board of Health and Welfare, patients must give their approval to be included in the NQRs. The NQR are used in clinical settings, management and research to improve delivery of health care [50].

Sweden has unique personal identification numbers that every person residing in Sweden receives as an identifier [51]. Every person keeps the same number throughout life. The number is used for communication between government authorities, private companies, other organizations, and citizens. It could also be used for research purposes as it allows linking of data from different registers to one specific individual.

All health care provided by Region Skåne and by private care providers under contract with Region Skåne is recorded in Skåne Health care register (SHR). This register includes data on all delivered care within Region Skåne since 1998 on an individual level. Each health care contact generates data entries by the health care provider that are transferred to this central database. Thus, the SHR contains data from visits to all primary care, outpatient specialized care and hospital care. Each record includes ICD-10 (International Statistical Classification of Diseases and Related Health Problems, 10th Revision) [1] diagnostic codes, treatment codes, date of visit, type of care, type of health care professionals. The register is connected to the reimbursement in Region Skåne and the coverage is therefore generally very high [52].

Whereas health care is provided on the regional level, the responsibility for social service and support lies on Sweden’s 290 municipalities. This includes service and support for people with permanent functional impairments according the Act concerning Support and Service for Persons with Certain Functional Impairments (the LSS act) [47]. This includes, for example, daytime activities, residential care, and relief services for next of kin. The goal of the LSS act is that people with functional impairments should be able to participate and be equal members in society and to live an ordinary life as others. The LSS act defines three broad groups of functional impairment, whereof one is those that have ID and/or ASD (Autism Spectrum Disorder) that have been present since birth or early years. The group affiliation and all provided support is recorded in the national LSS register, which is maintained by the Swedish National Board of Health and Welfare. Thus, it is possible to use the LSS register to separate people with ID and/or ASD from those with other kinds of functional impairments and those with intellectual functional impairment acquired in adult life, for example, caused by traumatic brain injury. However, it is not possible to make distinctions within groups, i.e., the register does not comprise information to separate people with ID from those with ASD. It is mandatory for all municipalities to submit data to the LSS register and The National Board of Health and Welfare retrieves data annually.

### Study description

IDcare is a register study, based on regional and national registers. All people living in Skåne on January 1st, 2014, were included. From these, we created three cohorts, the ID cohort (i.e., people with ID), the ID family cohort (people living in the same household as a person in the ID cohort), and the gPop cohort (general population). As this is a study based entirely on national- and regional registries, participants did not need to be recruited. However, we provided easy-to-read information about the study on a Lund University website (https://www.lupop.lu.se/idcare), offering the possibility to opt out.

### Study population

Through data from the Swedish total population register people living in Skåne on January 1st, 2014, were identified. The register contains information provided by the Swedish Tax Authority and comprise the civil registration of vital events such as deaths, marriage, and change of address. It comprises all people residing in Sweden and is maintained by Statistics Sweden.

People were included in the ID cohort in two different ways. People that during the study period (2014–2020) had at least one diagnosis of ID (ICD-10 codes F70-F79) or Down syndrome (DS; Q90) in the SHR and/or had support and/or services according to the LSS act and belonged to the ID/ASD group were included in the ID cohort. People living in the same family and/or household as someone in the ID cohort constituted the ID family cohort. The remaining comprised the general population cohort (gPop).

### Data collection and data management

#### Sociodemographic variables

We used various registers at Statistics Sweden to collect data on *sociodemographic variables*. Due to delays in registration of data, there is some variation in the years of data collection. We collected data about age, gender and place of living 2014–2021. We also obtained data concerning family/household, incomes (for the individual, the family, and the household; 2014–2020), employment status in November each year (2014–2019), and highest level of education (2014–2020).

#### Outcomes

Data concerning support and service *for people with certain functional impairments* according to the LSS act [47] was collected from the LSS register for 2007–2020.

SHR for 2014–2021. These included information on date, what kind of contact (e.g., physical visit or telephone call), whether they were planned or not, what kind of specialty and professional were involved, primary diagnosis and up to seven secondary diagnoses. As many variables in the crude data sets had high levels of detail we chose to aggregate to higher levels in our analyses. For example, we aggregated all types of physicians. This means that for example childcare centre physician, maternity care centre physician, pre-registration house officer, senior house office, gynaecologist and teams that included a physician were merged into the higher level “physician”. Similarly, “nurse” included for example childcare centre nurse, maternity care centre nurse, COPD nurse, diabetic nurse, district nurse, hypertension nurse, and teams without physicians. Furthermore, we defined contacts as either physical (new visit, revisit), or non-physical (contact through mail, email, digital meeting online etc.). As the crude data still contain the detailed variables, these could still be used for specific research questions.

Information on the date and *place of death, the age at death*, and the cause of death registered according to ICD-10 was collected from the Swedish Cause of death register. This register is maintained by the Swedish National Board of Health and Welfare and contains data on all deaths in Sweden.

Three additional registers were used to collect COVID-19-related data for the pandemic period (2020–2021).

The Swedish National Vaccination Register is a health data register maintained by the Public Health Agency of Sweden and used to assess effects of national vaccine programs. Since January 1st, 2021, it is mandatory for health care providers to report all vaccinations for COVID-19 to the register. From this register, we collected data regarding COVID-19 vaccinations, including the date of vaccination, and the type and batch number of the vaccine.

The Swedish Intensive Care Unit (ICU) register (SIR) is an NQR. We collected data from care episodes in the ICU regarding vital parameters and lab samples recorded on admission to the ICU, daily Sequential Organ Failure Assessment scores, a selection of pre-existing diagnoses, and care episode specific diagnoses. Moreover, a burden of care scoring consisting of eleven different parameters and measures the patient-specific workload for nursing staff, recorded three times per 24 h was collected.

The Swedish register of palliative care is an NQR that includes data about the care of people during their last week of life. Data are collected by a questionnaire which is answered by health care staff after the person has died. The collected data from this register comprised data about place of death, prescription of drugs against end-of-life symptoms, assessment of symptoms and information provided to the caretaker and next of kin.

For the first part of the project, a request was sent to Statistics Sweden to create a study cohort with pseudonymized data. Requests were also sent to National Board of Health and Welfare and Region Skåne to disclose socioeconomic- and health care utilization data respectively. Statistics Sweden coordinated the pseudonymization, merging, and delivery of the data using the personal identification number for every person in the study population. Moreover, family- and household-related data were linked to unique family and household identification numbers, respectively. When the project was expanded to include the years for the COVID-19 pandemic and the registers related to intensive care, vaccination and palliative care, the register holders waited for the additional ethical approval and requests concerning the remaining registers before delivering the data. Statistics Sweden will keep a key for the pseudonymization until 2025, thus enabling further addition of data.

## Cohort characteristics

The original study population, i.e., people living in Skåne January 1st, 2014, comprised 1 274 727 people. However, we excluded 1 368 people for whom the personal identification number could not be uniquely determined, leaving 1 273 359 people. Of these, 11 458 (0.90%) had at least one measure of support according to LSS, 9 390 (0.74%) had at least one F7-diagnosis, and 848 (0.07%) at least one Q90-diagnosis. These were included in the ID cohort, which comprised 14 716 people (Table 1 and 8 777 [60%] men and 5 939 [40%] women). The ID family cohort comprised 31 688 people (15 567 [49%] men and 16 031 [51%] women) who, based on the family and household identification numbers, belonged to the same family and/or household as a person in the ID cohort. The remaining people comprised the general population referent (gPop) cohort, which thus included 1 226 955 people (606 923 [49%] men and 620 032 [51%] women).

**Table 1:** People included in the ID cohort (*n* = 14 716), without and with LSS-support according to level of F7 and presence of Q90

	Total	No LSS-support	LSS-support
**F7 and/or Q90**			
F7 without Q90	9 024	3 163 (35%)	5 861 (65%)
Q90 without F7	482	44 (9%)	438 (91%)
F7 with Q90	366	51 (14%)	315 (86%)
No F7 or Q90	4 844	---	4 844 (100%)
Any F7	9 390	3 214 (34%)	6 176 (66%)
Any Q90	848	95 (11%)	753 (89%)
**Type of F7**			
Mild (F70)	3 952	1 781 (45%)	2 171 (55%)
Moderate (F71)	1 086	260 (24%)	826 (76%)
Severe (F72)	406	77 (19%)	329 (81%)
Profound (F73)	207	59 (29%)	148 (71%)
Other/unknown (F78/F79)	3 739	1 037 (28%)	2 702 (72%)

The number of people with and without LSS-support for the different combinations of ID/DS diagnoses is described in Table 1. People with at least one F7-diagnosis were categorized according to the most severe diagnosis. Thus, a person with a diagnosis of mild ID during one health care contact and moderate ID during another was considered as having moderate ID. Those without any specified (F70-F73) ID diagnoses, i.e., with only F78 (other) or F79 (unknown) were combined into one category as there is no severity indication in these diagnoses. Among the 5 651 people with a specified diagnosis, 42% (*n* = 2 355) at one point also had an unspecified diagnosis (F78 or F79).

All people were categorized by age at study start (i.e., 2014) into the categories children, adolescents, young adults, adults, lower middle-age, upper middle-age, older, and oldest old(Table 2). The reasons for this were to be able to investigate unique patterns of health and health care in specific age groups. For example, health care use among the youngest age groups is likely to be marked by their parents and many adolescents, that in the beginning of the study period receive health care within paediatric care, will, when they become young adults, be transferred to general health care. The number of people in the different age categories in gPop and the ID cohort respectively is presented in Table 2.

**Table 2:** Number of people in different age categories and cohorts

	Age	Year of birth	gPop	ID	ID family
Children	0–12	2002–2014	171688 (14%)	3565 (24%)	5978 (19%)
Adolescents	13–18	1996–2001	73081 (6%)	2072 (14%)	3399 (11%)
Young adults	19–24	1990–1995	96811 (8%)	2380 (16%)	2979 (9%)
Adults	25–44	1970–1989	324553 (26%)	3902 (27%)	9546 (30%)
Lower middle-age	45–54	1960–1969	160913 (13%)	1147 (8%)	5603 (18%)
Upper middle-age	55–64	1950–1959	143057 (12%)	884 (6%)	2607 (8%)
Older	65–79	1935–1949	183875 (15%)	659 (4%)	1285 (4%)
Oldest old	≥ 80	< 1935	72977 (6%)	107 (1%)	291 (1%)

### Including people with ASD

As LSS support is provided to people with ASD as well as people with ID, and no information on diagnosis is available in the LSS register, we cannot rule out that people with ASD but without ID have been included in the ID cohort. In the study population as a whole, 85% of those with diagnosis of ASD did not have a concomitant diagnosis of ID. The corresponding number among those with LSS support was 67%. The number of people included in the ID cohort based on LSS support only, i.e., where no diagnosis of ID was found, was 4 844. Assuming that the distribution of ASD and ID among those with LSS support only is similar to that in the whole study population and those with LSS support, respectively, the number people in the ID cohort with diagnosis of ASD and not ID would be 4 117 (based on 85%) and 3 245 (based on 67%). This corresponds to 28% and 22% of the ID cohort, respectively. The percentage of possibly misclassified decreased with age group (Table 3).

**Table 3:** The proportion of people with Autism spectrum disorder (ASD) with and without LSS support in the population and estimated in the ID cohort

			ASD without ID			
			Whole study population		With LSS support	
	ID cohort	LSS support only	Actual in population	Estimated in ID cohort	Actual in population	Estimated in ID cohort
Children	3565	1157	87%	1007 (28%)	68%	787 (22%)
Adolescents	2072	825	85%	701 (34%)	69%	569 (27%)
Young adults	2379	895	81%	725 (30%)	69%	618 (26%)
Adults	3902	1394	84%	1171 (30%)	69%	962 (25%)
Lower middle-age	1147	271	78%	211 (18%)	55%	149 (13%)
Upper middleage	884	176	70%	123 (14%)	40%	70 (8%)
Older	659	108	64%	69 (10%)	41%	44 (7%)
Oldest old	107	18	40%	7 (6%)	0%	0 (0%)
All	14,715	4844	85%	4117 (28%)	67%	3245 (22%)

### Potential exclusion from the ID cohort of people with ID but without LSS support or diagnosis

**Fig. 1:**
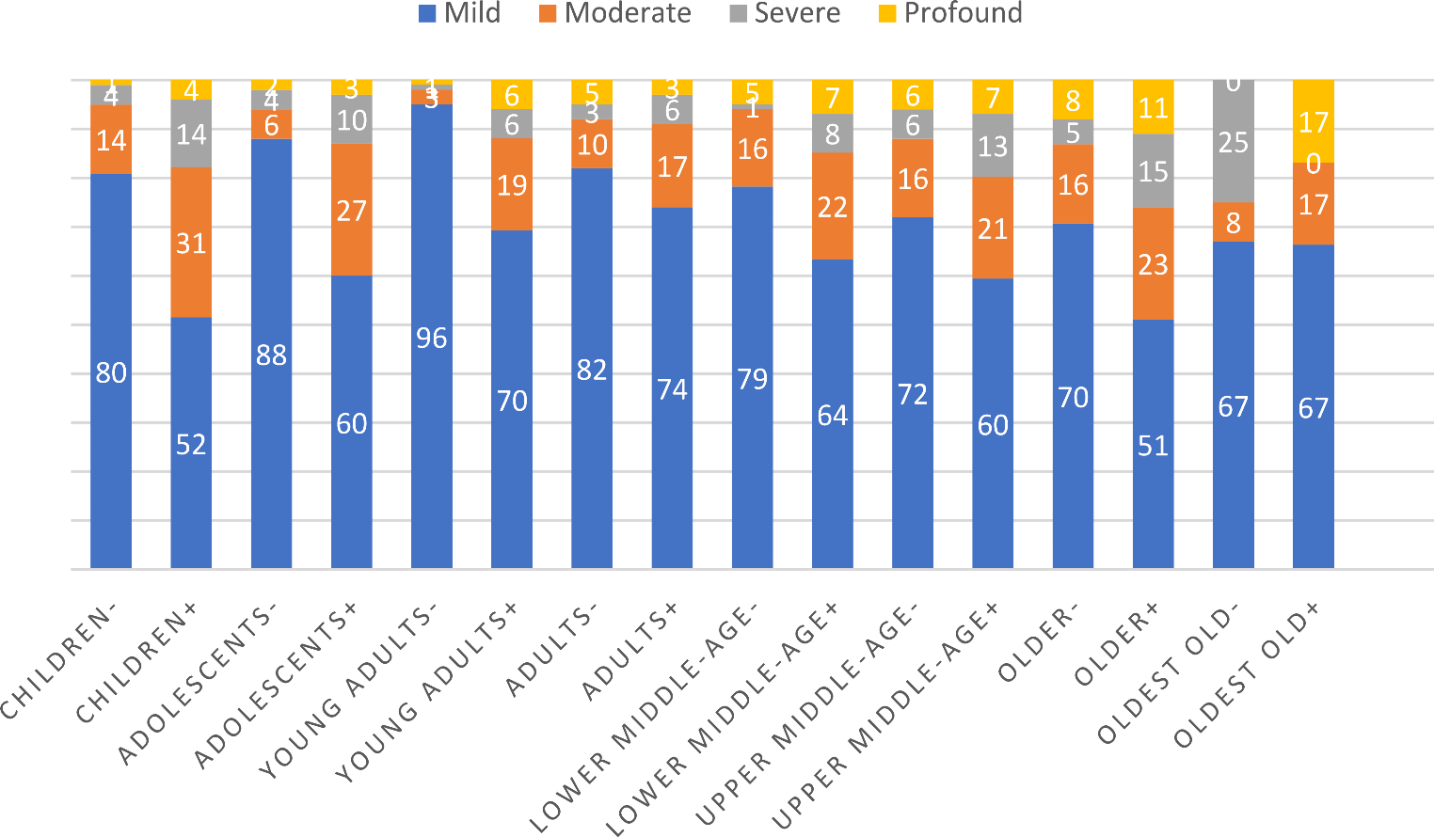
Difference in distributions of severity of ID among those with (+) and without (-) LSS support, in different age groups. Statistically significant differences were found for children (*p* < 0.001), adolescents (*p* < 0.001), young adults (*p* < 0.001), adults (*p* < 0.001), and lower middle-age (*p* = 0.005), but not for upper middle-age (*p* = 0.255), older people (*p* = 0.083), and the oldest old (*p* = 0.290)

People with ID but without diagnosis or LSS support during the study period (2014–2021) were not included in the ID cohort. Among those with an ID diagnosis, severity of ID (not including those with other/unspecified ID) was differently distributed among those with and without LSS support, with severity of ID being more skewed towards mild among those without LSS support than those with LSS support (*p* < 0.001, Pearson chi-square). The shift towards more mild ID was more pronounced in the younger age groups (Fig. 1). However, in the older age groups, ID diagnosis with-out specification of severity (i.e., other/unspecified ID) was more common than in the younger age groups (13% among children, 20% in adolescents, 42% in young adults, 53% in adults, 57% in lower middle-age, 66% in upper middle-age, 74% in older people, and 78% in the oldest old). To understand the meaning of the diagnoses other and unspecified ID, we investigated those with at least one such diagnosis. Among those with at least one diagnosis of other ID (F78), 21% also had at least one diagnosis of mild ID, 10% had a diagnosis of moderate ID, 4% of severe ID, and 3% of profound ID. Among those with at least one diagnosis of unspecified ID (F79), 23% also had a diagnosis of mild ID, 9% had a diagnosis of moderate ID, 5% of severe ID, and 2% of profound ID. The shift towards milder ID among those without LSS and among those with diagnosis of other/unknown ID supports the conclusion that people with mild ID are less likely to have LSS support. This is not surprising, as they may very well be able to handle their daily activities to a much greater extent than those with more severe ID. Thus, the people we failed to include in the ID cohort due to lack of diagnosis or LSS support during the study period probably have milder forms of ID.

### Strengths and limitations

The creation of this cohort has both strengths and limitations. The main strength is the comprehensiveness in the number of participants as all people of all ages living in the county of Skåne is included. Previous studies are in general based on small samples and certain age groups [36–41]. The use of the whole population as well as the exclusion of those living in the same family as those with ID decrease the risk of selection bias. Another strength is the longitudinal design and that data for all health care contacts during the study period, both before and during COVID-19, is collected. In Sweden, all health care contacts are registered as they are connected to the reimbursement system for the health care providers. The national patient register (which SHR is reporting to) [53] as well as the SHR itself [52] have been found to have high validity. A third strength and what makes this study unique is that we used two different registries to identify our ID cohort, both diagnosis in SHR and service and support according to LSS. A potential weakness is the use of service and support for people with ID and/or ASD as an inclusion criterion for the ID cohort, which could have contributed to selection bias. Another potential weakness is that some care providers have poorer compliance when it comes to registration of diagnoses, and that diagnoses from private facilities are not available. Furthermore, it is important to acknowledge that despite having all diagnoses for eight years it is possible that people in the sample have been diagnosed before the study period. It is also possible that people in the sample have a disease that has not been diagnosed or registered in SHR. This means that the any prevalences are probably underestimated. But, as this is true for both groups, the data is still valid for comparisons.

### Ethics

People with ID constitute a vulnerable group in society. Establishing a cohort comprising people with ID and linking sensitive data such as that concerning health and health care utilization, must be done with caution. Therefore, we applied for approval from the Swedish Ethical Review Authority (dnr 2021 − 01444 with amendments 2021–04910 and 2021–06056). Moreover, the Swedish National Board of Health and Welfare and Statistics Sweden, performed their own secrecy review before providing the data.

At the same time, given the availability of register data on health care, service and support, and causes of death in combination with the suspicion that the health care needs of people with ID may have been given low priority during the pandemic and the already established cohorts of people with ID and referents from the general population, one may argue that it would be unethical *not* to wider explore potential inequalities within the welfare state.

### Collaboration

IDcare comprises data from several national and regional registers that have been merged into one database. This database comprises a large amount of personal information on an individual level. This means data is detailed enough to enable identification of, at least, some of the people included. Therefore, these data cannot be made publicly available. However, as the database was compiled by public register data, other researchers may generate an identical database by contacting the register holders and thereby get access to the registries used in this study.

The researchers will collaborate with researchers that are experts in the field of ID and/or who could contribute by interpreting the results in the different domains that will be investigated, for example health care utilisation or diagnostic panoramas. Collaborators will, if willing, be listed on the IDcare website (https://www.lupop.lu.se/idcare).

Table 1 People included in the ID cohort (*n* = 14 716), without and with LSS-support according to level of F7 and presence of Q90.

## Data Availability

Since the data comprise information about single individuals and is detailed enough to enable identification of, at least, some of the people included, they cannot be made publicly available. However, as the database was compiled by national register data, other researchers may contact the register holders to get access to the registries used in this study, and thereby generate an identical database.
